# Complexity Meets Risk—The Next Generation of Genome-Edited Plants Challenges Established Concepts for Environmental Risk Assessment in the EU

**DOI:** 10.3390/plants14111723

**Published:** 2025-06-05

**Authors:** Marion Dolezel, Marianne Miklau, Andreas Heissenberger, Iris Kroeger, Mathias Otto

**Affiliations:** 1 Land Use & Biosafety Unit, Umweltbundesamt–Environment Agency Austria, Spittelauer Laende 5, 1090 Vienna, Austria; marianne.miklau@umweltbundesamt.at (M.M.); andreas.heissenberger@umweltbundesamt.at (A.H.); 2 Division Assessment Synthetic Biology, Enforcement Genetic Engineering Act, Federal Agency for Nature Conservation, Konstantinstrasse 110, 53179 Bonn, Germany; iris.kroeger@bfn.de (I.K.); mathias.otto@bfn.de (M.O.)

**Keywords:** genetically modified plant, genome editing, complex modification, GMO, environmental risk assessment, comparative safety assessment, protection goal

## Abstract

For 20 years, the environmental risk assessment (ERA) of genetically modified plants (GMPs) has used a comparative assessment approach, comparing the GMP to presumably safe and familiar non-modified plant varieties. With new genomic techniques, it is now possible to design complex GMP applications with systemic metabolic changes, resulting in novel plant phenotypes. These plant phenotypes can exhibit profoundly altered morphological, physiological, or compositional characteristics, intentionally lacking equivalence with parental plants and non-modified comparators. Through the analysis of case studies involving GMPs with modifications of complex metabolic pathways, we evaluate the current practice of the comparative safety assessment approach applied in ERA in the European Union and its ability to inform ERA, particularly regarding environmental risks. Our findings show that the existing approach has notable weaknesses when applied to complex GMP applications. We suggest complementing ERA with a hypothesis-driven assessment approach that considers various protection goals and relies on whole-plant experimental assessments to draw risk conclusions. As plant modifications become increasingly complex, such as the development of synthetic biology plants, conducting ecologically realistic assessments will be crucial for future ERA.

## 1. Introduction

With the emergence of new genomic techniques (NGTs) for plant biotechnology, specifically CRISPR/Cas9, a range of novel applications of genetically modified organisms (GMOs) and, specifically, genetically modified (GM) plants (GMPs) are currently being developed [1]. By using NGTs in plant breeding, intervention into the plant genome can be considerably extended due to the simplified accessibility of the whole genome and the possibility to simultaneously edit or knockout multiple genomic loci, which is also referred to as the multiplexing of genomic modifications [2,3,4,5,6,7,8]. So far, more than 100 genes have been simultaneously modified [9]. This multiplexing process has been applied to develop GMPs with enhanced biotic (e.g., pathogen) or abiotic (e.g., drought) stress tolerances, or combinations of the two [10,11,12,13]. In addition, GMPs with extensive and multiple morphological changes, e.g., plant development, plant growth, seed shattering, flowering, grain size, or inflorescence architecture, have also been developed (see examples in [14,15]). Specifically, the modification of complex plant metabolic pathways has become a new field of research. Inter alia, the metabolic engineering of plants aims to produce certain substances in crop plants through the modification of several connected genes of metabolic pathways or even whole gene networks. Many of these complexly modified plants have also been termed “synthetic biology plants” [16,17,18]. In 2014, the European Commission’s Scientific Committees presented an opinion on synthetic biology [19] that recognized the emergence of new agri-food applications in relation to synthetic biology, stating that they would require prior authorization in Europe. Based on this opinion, the European Food Safety Authority (EFSA) recently evaluated the implications for the risk assessment of GMOs developed using synthetic biology approaches [20].

The environmental release and use of GMPs is regulated across the world, and authorizations are generally based on risk assessments [21,22]. In the European Union (EU), the market approval of GMPs, which includes genome-edited plants and NGT applications, is linked to an obligatory ex ante assessment of risks to human and animal health, as well as the environment, according to Directive 2001/18/EC, Directive (EU) 2018/350, and Regulations (EC) No. 1829/2003 and No. 503/2013. These EU regulatory provisions acknowledge that GMPs, which can spread and reproduce in the environment, may have effects that are irreversible (Recitals 4 and 5, Directive 2001/18/EC). For these reasons, the provisions are firmly grounded in preventive action and the precautionary principle (Recital 8, Dir 2001/18/EC). These normative foundations are reflected in the mandatory case-by-case risk assessment and stepwise release of GMPs into the environment (using a step-by-step approach).

A general principle in the environmental risk assessment (ERA) of GMOs is the comparison of the GMP with the non-modified plant from which it is derived in order to identify potential adverse effects arising from the genetic modification (Directive 2001/18/EC, Annex II). In the problem formulation step of ERA, (i) any changes in the characteristics of the organism, which are linked to the genetic modification, and (ii) potential adverse effects on human health or the environment linked to the identified changes should be assessed (Directive (EU) 2018/350, Annex C.3). This principle is further outlined in guidance documents for ERA published by the European Food Safety Authority (EFSA) [23,24,25]. In addition to using the non-GM parental line as a comparator, EFSA recommends using a range of reference plant varieties for the comparison of compositional, agronomic, and phenotypic plant characteristics [23]. The safety of the GMP can be concluded if no statistically significant differences or non-equivalences between the GMP and the non-modified comparators are detected, in addition to the separately assessed genetically modified trait [23,24]. This approach, which is used to derive the safety of the GMP by comparison to several non-modified plant varieties, is referred to as the “comparative safety assessment” by EFSA.

Comparing complex GMP applications to conventional, non-modified plant species can be challenging due to the extensive changes in compositional, physiological, or morphological characteristics. Although limitations of the existing comparative assessment approach to assess food and feed safety, as well as environmental safety, have been recognized for such applications [26], no other risk assessment approaches (e.g., assessments without comparators) have been applied so far in the ERA of GMPs. For the food and feed safety assessment of GMPs, including those with complex traits, regulatory authorities worldwide have relied on a comparative assessment, although slightly different methods have been used [27]. For example, when conducting risk assessment of GMPs with novel traits, regulatory bodies in the US, Canada, Australia, and New Zealand use a comparative approach, despite substantial differences between the GMP and the conventional plant, such as in food safety-relevant compounds [27]. In the case of complex changes in plant composition or the lack of safe comparators, the EU regulatory provisions for GM food and feed safety (Implementing Regulation (EU) No 503/2013) foresee a stand-alone risk assessment as required for novel foods (according to Regulation (EC) No 258/9) to conclude on safety. Hitherto, similar provisions for the assessment of the environmental safety of GMPs with complex traits are lacking.

Recently, EFSA recognized the limits of the comparative safety assessment approach for GMPs obtained through synthetic biology [10]. This was exemplified by the case study of a de novo domesticated tomato, for which EFSA suggested applying either a stand-alone assessment or using several—so far undefined—comparator lines for risk assessment. The further development of the comparative safety assessment or the application of alternative assessment approaches was recommended, together with an update of the existing guidance for ERA [10,20]. Alternative concepts have been proposed for the risk assessment of different environmental stressors, including GMPs [28]. However, so far, applicants have refrained from implementing such concepts for the risk assessment of GM crops.

The purpose of this article is to analyze whether the current concept of a comparative safety assessment applied in ERA is practicable for GMPs with complex genetic modifications. There is currently no universally accepted definition of complexity regarding genetic modifications. However, a common characteristic of complex systems is the non-linearity of interactions among system components, including living organisms interacting with the environment, which we find particularly relevant in the context of complex GMP applications (for further discussion on complexity see, e.g., [29]). Therefore, we specifically consider GM approaches, where modifying a specific component is likely to impact upon many other components and their interactions. We define the complexity of a GMP application at various levels, i.e., the level of genetic modification, the level of the modified trait, or the breeding level.

Complexity in genetic modification refers to the genetic engineering process of complex interactive networks, particularly the multiplexing of knockout mutations or other editing steps involving multiple alleles, multiple gene copies, or multiple genes, resulting in new plant genotypes with multiple mutations in one generation [5,6,7,8,9,10,11,30]. Although such complex modifications are particularly common in applications for industrial purposes, they are also used to achieve biotic and abiotic stress tolerance in plants [3]. One specific example is the utilization of transcription factors for genetic modification. Transcription factors (TFs) are DNA-binding proteins that serve as primary regulators of transcription and are part of a network of molecular regulatory elements that govern gene expression. In plants, TFs are extensively studied for genetic crop improvement, specifically to achieve traits like biotic and abiotic stress tolerance [31]. This also involves modulating gene expression by targeting regulatory elements for transcriptional regulation [7,9]. Research in this area includes modifying rate-limiting enzymes while simultaneously blocking branch points in metabolic pathways or activating key enzymes through TFs [32].

Complexity at the trait level refers to applications involving modifications of quantitative trait loci (QTL), such as multi-gene traits that result in physiological or phenotypic changes or altered responsiveness to environmental cues [2,30,33]. Often, key enzymes in metabolic networks are targeted, such as those involved in photorespiratory pathways [34], fatty acid synthesis [35], plant hormone synthesis [15], or secondary metabolite pathways [36]. This encompasses traits that impact nutrient uptake or nutrient use efficiency, traits that enhance adaptation to changing environmental conditions (e.g., drought tolerance), and novel mechanisms for pathogen defense. Additionally, the engineering of entire metabolic pathways to produce novel and new-to-nature metabolites or de novo proteins with novel biological functions is also included [10,20,37].

Complexity at the breeding level refers to the complexity involved in plant breeding schemes. This includes GMP applications that have been developed by combining (i.e., stacking) single-trait GM events through conventional breeding, resulting in multiple GM events combined in one GM plant [33]. It also includes the use of techniques developed to facilitate complex breeding schemes, e.g., haploid induction and reverse breeding [2], the use of synthetic biology approaches in breeding [37], and de novo domestication methods [10].

In this article, we first outline the current practice of ERA for GMPs based on the comparative safety assessment. We explain the concepts underlying the currently used assessment approach by referencing well-known examples of classical GMPs and discussing its pitfalls in ERA. Next, we address the limitations of the comparative safety assessment approach for complex GMP applications using selected examples. We have chosen examples of GMPs with complex modifications, as outlined above, that are currently being discussed in the scientific literature or have already been reviewed by EFSA (Table 1). Finally, we propose the necessary conceptual and methodological adaptations required to evaluate the potential environmental effects of complex GMPs in ERA. We conclude that assessing the environmental risks of complex GMP applications should not rely solely on the results of a comparative assessment of standard plant characteristics with other plant varieties. Instead, we suggest additional assessment approaches based on focused risk hypotheses that are relevant to protection goals.

## 2. The Current ERA Practice in the EU

### 2.1. Consideration of Intended and Unintended Changes in the GMP

**Figure 1:**
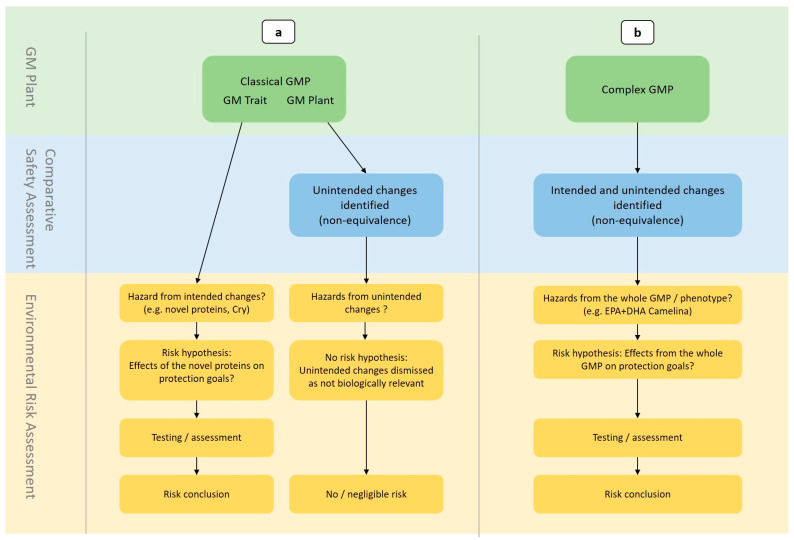
Environmental risk assessment (ERA) of classical GMPs (**a**) and complex GMPs (**b**). For classical GMPs, non-equivalences between the GMP and the non-modified plant identified in the comparative safety assessment are generally dismissed in ERA; only hazards due to the intended traits are assessed (**a**). For complex GMPs, hazards due to the whole phenotype need to be assessed in ERA based on potential risks to protection goals (**b**). See text for further explanation.

In ERA, any type of change occurring in the GMP needs to be identified and characterized in view of its potential to impact human and animal health or the environment. This includes both intended and unintended changes in the plant, which may result from genetic modification. While intended changes refer to “…changes that are designed to occur and which fulfill the original objectives of the genetic modification”, unintended changes are “…consistent changes which go beyond the intended change(s) resulting from the genetic modification” [24]. During the ERA of classical GM crops such as herbicide-tolerant soybean or insect-resistant maize, the intended change typically involves the expression of one or a few new proteins that have not been previously expressed in the plant. The newly introduced GM trait, i.e., the 5-Enolpyruvylshikimate-3-Phosphate-Synthase (EPSPS) protein for herbicide-tolerant crops or a *Bacillus thuringiensis* (*Bt*) protein for insect-resistant crops, is assumed to be the only difference between the GMP and the non-GM comparator. Therefore, it is considered to be the only stressor to be evaluated in terms of potential risks to relevant protection goals. Based on the knowledge of the potential effects of novel toxins (e.g., the cross-order toxicity of Cry-toxins), specific risk hypotheses for the potential effects of these newly expressed proteins on protection goals are formulated in ERA. This includes the potential toxicity of the Cry-toxin to humans, animals (e.g., livestock), or non-target organisms that may be exposed to the GMP. Following a tiered testing approach, the potential hazards of the GM trait, i.e., the newly expressed proteins, for these organisms are evaluated (Figure 1a).

This approach is based on the assumption that the non-GM plant (i.e., the parental plant), which was used for genetic modification, is familiar and has a history of safe use (HoSU). Therefore, no unintended changes are expected to occur in the plant. However, in addition to the intended changes caused by the GM trait, it is also possible that unintended changes resulting from the genetic modification need to be addressed in ERA. Such unintended changes may occur due to the heterologous expression of proteins in transgenic plants, which can affect the plant’s metabolism. For example, the overexpression of EPSPS genes, as observed in herbicide-tolerant crops, can affect endogenous plant hormone levels (e.g., auxin levels) and, consequently, the plants’ fecundity and fitness [38]. In other cases, the observed changes can be due to position or pleiotropic effects of the transgene insertion or the transformation method itself [39]. Also, new genomic techniques (e.g., CRISPR/Cas9) applied to achieve targeted mutagenesis in plants have the potential to introduce unintended changes at the molecular level, which can also result in unintended phenotypic changes [39,40,41]. In transgenic crops, the occurrence of unintended metabolic inferences has been described as beneficial, especially when they result in stress-tolerant phenotypes [39,42]. Such changes in the plant genome and, consequently, metabolome are largely unpredictable due to a lack of knowledge with respect to the interactions between the newly expressed heterologous protein(s) and the specific metabolism of the modified plant. This hampers the formulation of specific risk hypotheses in ERA (Figure 1a).

### 2.2. Comparing GMPs with Presumably Safe Plants—History of Safe Use and Familiarity

As a solution, the comparative safety assessment approach has been established by EFSA. This approach requires a comparison of the GM plant with its conventional (non-GM) counterpart, as well as with other non-GM comparator varieties in various predefined compositional, agronomic, and phenotypic characteristics [43]. These are usually assessed in agronomic field trials in representative environments [24,43]. The results of the assessments are then evaluated by two complementary statistical tests that need to be carried out by the applicants. The difference test aims to verify whether the GMP is different from its comparator. In addition, the equivalence test should verify whether the characteristics of the GMP fall within the range of natural variation, which is estimated from the set of non-GM reference varieties with a history of safe use, which were simultaneously grown with the GMP in the field trials [33,44]. The outcomes of these two statistical tests then determine whether the GMP is equivalent to the non-GMP, and further structure the risk assessment [23,33,45].

The comparison of the GMP with a conventional counterpart to determine its safety is closely interlinked with the concept of the History of Safe Use (HoSU). This concept refers to conventional food plants, i.e., edible varieties of the same species used as comparators for GMPs [46]. In food and feed risk assessment, the HoSU concept is combined with the concept of substantial equivalence [33]. Thereby, a “normal range” of analytes for selected food components such as key nutrients, anti-nutrients, or secondary plant metabolites is defined for traditional crop plants like potatoes, soybeans, or maize. Any compositional difference between the GMP and the conventional food plant is put into context with the variability observed in other varieties [46,47]. Regulation (EC) No. 1829/2003 and Implementing Regulation EU (No.) 503/2013) lay down the principles for the authorization of genetically modified food and feed; both refer to the HoSU approach for ERA purposes. However, the exact definitions of HoSU are lacking, such as the time period or level of consumption that indicates safe use [26]. Similarly, for the concept of substantial equivalence, no safety limits have been defined that could aid in the interpretation of observed differences [48]. As an OECD concept, risk assessors worldwide rely on the concepts of HoSU and substantial equivalence, although the extent and interpretation of these concepts may vary (see e.g., [27,49,50]). For the assessment of environmental risks, instead of the HoSU concept, EFSA refers to the concept of familiarity [24]. The US National Academy of Sciences originally introduced the concept of familiarity [51], referring to the knowledge on the biology of and experience with a traditionally cultivated plant used for comparison with a GMP [24]. The concept was initially developed for small-scale releases of GMPs, considering that potential hazards were foreseeable and manageable based on previous experience with traditional crops [52]. The OECD further extended the concept to large-scale releases of GMPs [53]. The criteria for familiarity include knowledge about and experience with the crop plant, the environment, the trait, previous genetic modifications of the crop, and crop–trait interactions with the environment [53]. Based on this concept, any non-GM crop plant is presumed to be safe and, therefore, the accepted norm to which the GMP is compared. The application of the familiarity concept for current ERA was criticized as being too focused on agricultural aspects [54]. Other authors considered that the concept accepts the potential adverse indirect effects of the GMP, such as effects due to cultivation methods (e.g., herbicide use), if these are considered familiar with a certain (non-GM) crop [55]. Consequently, the familiarity concept evaluates whether risks are foreseeable and manageable, but does not assume safety per se.

### 2.3. Pitfalls of the Comparative Safety Assessment Approach in ERA

So far, all GM crops notified for import (food and feed purposes, excluding cultivation) in the EU according to Regulation (EC) No. 1829/2003 have been risk assessed by use of the comparative safety assessment approach [37]. In general, any observed differences and non-equivalences between the GMP and the non-modified plants in compositional, agronomic, or phenotypic parameters are evaluated with respect to their implications for food and feed safety. This approach is useful for food–feed risk assessment, if reference plant varieties with a history of safe use are available [56] and if parameters are assessed with known relevance for the protection goal relating to human and animal health (e.g., toxicology or allergenicity). In ERA practice, the approach shows several pitfalls. The assessed parameters are derived from various OECD documents outlining food–feed-relevant nutrients, anti-nutrients, toxins, or allergens for a range of crop plants, e.g., oilseed rape or soybean [57,58]. However, even in the case of observed differences or non-equivalences between the GMP and its non-GM counterpart or the conventional plant varieties, EFSA has so far accepted the applicant’s argumentation that the differences were within established ranges of natural variability and did not affect the food and feed safety of the GMP. So far, no biological relevance or even risk has been assigned to any observed changes, and further assessments were not considered necessary by EFSA. Therefore, the potential differences or non-equivalences are typically not seen as indicators of unintended metabolic changes in the GMP and do not lead to further assessments (Figure 1a).

#### Example: GM High Oleic-Acid Soybean

The pitfall of this approach becomes evident with the modification of metabolic pathways in oilseed plants. An example is GM soybean *Glycine max*, which has been notified for import and processing into the EU (event 305423). The GM soybean expresses a fragment of the *fad* gene, leading to the silencing of the plant’s endogenous *fad* gene (through the use of RNAi), resulting in a decreased level of the ω-6 fatty acid desaturase and, consequently, a high oleic-acid phenotype. The fatty acid composition of the seeds of the GMP showed several significant differences and non-equivalences when compared to the non-GMP. In total, 51 parameters in seeds were significantly different and for 16 of these, equivalence could not be established [56,59,60]. Thirteen statistically significant different fatty acid values between the GM soybean and the conventional counterpart were detected, and the equivalence test indicated that equivalence between the GM and the set of commercial varieties was less likely than not (category III) or demonstrated non-equivalence (category IV; see Table 1 in [60]). As intended, the levels of the targeted fatty acid (oleic acid) increased more than threefold (from 20% to 70%). In addition, other mono-unsaturated and saturated fatty acids increased in abundance. Specifically, the levels of so-called “intermediate” fatty acids, heptadecanoic acid (HA, C17:0) and heptadecenoic acid (HE, C17:1), increased approximately sevenfold and twentyfold, respectively. Simultaneously, levels of polyunsaturated fatty acids such as linoleic and linolenic acid (C18:2 and C18:3) decreased by about 90% and 40%, respectively. Other constituents in the GMP were also non-equivalent, such as the levels of trypsin inhibitor, which decreased by about 50%, but also those of zinc, calcium, and the isoflavone glycitin. In its scientific opinion, EFSA recognized that the composition of the GM soybean differed from the conventional counterpart and the non-GM reference varieties in its fatty acid profile but considered the observed non-equivalent levels of the odd-chain fatty acids HA and HE of GM soybean as unintended changes that were consistent with the objective of the genetic modification [60]. In all cases, the authority did not consider the changes in the fatty acid levels and the decreased level of trypsin inhibitor as a relevant food safety concern and required no further studies to follow up the observed results [60].

A similar problem as observed in food–feed risk assessment arises when the results of the comparative assessments are interpreted with respect to environmental safety. In these assessments, the plant characteristics of agronomic interest—such as yield or plant height—are measured under agronomic growing conditions and compared to a range of non-GM reference varieties. The results of these assessments (differences and non-equivalences) are then used to demonstrate the environmental safety of the GMP, e.g., with respect to changed ecological interactions or the potential for persistence and invasiveness in natural habitats. For the assessment of environmental risks of GMP applications that include cultivation in their scope, the use of reference values from conventional plant varieties to determine the biological relevance of the observed differences between the GMP and its non-GM counterpart is of limited use [61]. The shortcomings of this approach for ERA, specifically with respect to the test design and endpoints assessed, has been criticized elsewhere [62]. Instead, EFSA and others suggested the definition of minimum ecological effects that are considered relevant to cause harm, i.e., Limits of Concern [24,61]. The idea to derive such limits, which indicate harm if exceeded, was originally introduced by EFSA in its ERA guidance [24], but has not yet been implemented in practice [63,64,65].

## 3. Limitations of the Comparative Safety Assessment Approach for Complex GMP Applications

### 3.1. Complex GMP Applications Lack Equivalence with Non-GM Plants

In many complex GMP applications, several key steps in a metabolic pathway are modified to achieve the intended phenotype. These metabolic alterations can have profound implications for intermediary and interconnected metabolisms in the plant. Consequently, unintended changes in the plant phenotype with respect to composition, physiology, or morphology can occur, leading to a lack of equivalence between the GMP and the non-GM counterpart. In a comparative safety assessment, these changes are likely to result in significant differences and non-equivalent outcomes of the analyzed plant parameters. In the ensuing section, we present six categories of complex GMP applications to exemplify this point (Table 1).

**Table 1:** Complex GMP applications, their parental plants, intended GM traits, (unintended) phenotypic changes in the GMP, and potential environmental risks. GM = genetically modified; GMP = genetically modified plant.

No.	Complex GMP Applications	Parental Plants	Intended GM Trait(s)	(Unintended) Changes in the GMP	Potential Environmental Risks	References
1	Modification of endogenous seed metabolites	Various oilseed crops, e.g., oilseed rape (*Brassica napus*), soybean (*Glycine max*), camelina (*Camelina sativa*), safflower (*Carthamus tinctorius*), pennycress (*Thlaspi arvense*), field cress (*Lepidium campestre*)	Reduction in unwanted fatty acids (e.g., PUFAs, erucic acid) Enrichment of mono-unsaturated fatty acid (e.g., oleic acid) Increased TAG (triacylglycerol)-oil production Modification of seed coat color	Changes in relative frequency of various (e.g., medium chain) fatty acids Increased PUFA levels Changes in plant growth and development Changes in germination characteristics	Risks for trophic food webs and biodiversity Changes in plant survival and environmental interactions due to changes in biotic or abiotic stress tolerance Risks due to persistence and invasiveness due to changes in seed germination ability	[66,67,68,69,70,71,72,73,74]
		Cereals (e.g., *Triticum* sp.)	Modification of seed protein composition, i.e., change in gluten components (e.g., decrease in α, γ gliadins, in-or decrease in ω-gliadins)	Lower gliadin-to-glutenin ratio Production of novel α-gliadins Increased lysine contents		[75]
2	Production of novel fatty acids and oils in seeds	Camelina (*Camelina sativa*) Crambe (*Crambe abyssinica*)	Production of long-chain polyunsaturated fatty acids (LCPUFAs, “fish oils”) Production of wax esters (fatty alcohols)	Changes in overall fatty acid composition of seeds Production of intermediate fatty acids		[35,76,77,78,79,80,81,82]
3	Production of pharmaceuticals or nutraceuticals	Medicinal herbs, e.g., opium poppy (*Papaver somniferum*), Madagascar periwinkle (*Catharantheus roseus*) Food crops (e.g., tomato, rice)	Changed levels of endogenous alkaloids Expression of taste-modifying proteins Production of novel carotenoids (e.g., astaxanthin, canthaxanthin)	Production of various carotenoid intermediates	Risks for trophic food webs and biodiversity Risks for trophic food webs and biodiversity	[83,84,85,86,87,88]
4	Plants producing new-to-nature substances	Tobacco (*Nicotiana benthamiana*)	Production of novel biopesticidal molecules (e.g., crucifalexins, chlorobrassinin, bromobrassinin)	Not indicated	Risks to food webs and biodiversity due to novel biotic stress tolerance	[89]
5	De novo/re-domesticated plants	Wild plants, weeds, e.g., wild rice *Oryza alta*, barnyard grass *Echinocloa crus-galli*, *E. oryzicola*) Crop progenitors, e.g., wild tomato (*Solanum pimpinellifolium*) Ancient or orphan crops, e.g., ground cherry *Physalis pruinosa*, wild rice *Zizania latifolia*)	Modification of key domestication traits (e.g., shoot architecture, fruit characteristics, nutrient content, flower production, day-length sensitivity)	Not indicated	Risks due to persistence and invasiveness of new plants in novel environments or new cultivation techniques	[90,91,92,93,94,95,96,97]
6	Modification of photosynthetic pathways for more efficient carbon fixation (“green carbon plants”)	Model plants (e.g., *Nicotiana tabacum*, *Arabidopsis* sp.)	Increase in carbon assimilation, growth parameters (leaf area, number), biomass yield, light use efficiency	Decreased water use efficiency Changes in plant growth and development	Risks due to persistence and invasiveness due to changes in plant competition	[34,98,99,100]

#### 3.1.1. Plants with Modifications of Endogenous Seed Metabolites

In recent years, the genetic modification of oilseed crops to achieve changes in the fatty acid composition of seeds, using either classical transgenesis or new genomic techniques, has been extensively researched (see overview in [101]). Different oilseed crop species such as oilseed rape (*Brassica napus*), camelina (*Camelina sativa*), safflower (*Carthamus tinctorius*), soybean (*Glycine max*), pennycress (*Thlaspi arvense*), and field cress (*Lepidium campestre*) are targeted, and some of these are already commercially available outside the EU (e.g., high oleic-acid soybean and safflower). The modifications aim to reduce or eliminate unwanted fatty acids such as polyunsaturated fatty acids (PUFAs) or the enrichment of mono-unsaturated fatty acids, specifically oleic acid, for increased processing stability and shelf life. The modifications are usually achieved by a knockout of several genes that are responsible for fatty acid chain-length elongation or (de)saturation (e.g., fad genes) [66,67,68]. With the modification of these enzymatic genes to alter the fatty acid synthesis pathway, several changes in the composition, as well as the morphology and development of the modified plants, are commonly observed. For example, the knockout of *fad* genes in oilseed crops led to increased oleic acid levels (up to a maximum of 85%), accompanied by considerable changes in fatty acid profiles (e.g., linoleic acid) as well as alterations in several plant characteristics relevant for plant development and shoot architecture [66,67,69,102].

Another example is the modification of seed proteins in cereals (e.g., wheat) with the aim of reducing the gluten content of the seed. This was achieved by the downregulation of the endogenous seed gluten content by means of RNAi technology or genome editing [75,103,104,105]. The results showed an overall modification of protein composition in the mutated plant lines. The gluten proteins α- and γ-gliadin decreased significantly (by a maximum of 94%), while ω-gliadins varied extremely, with either no changes, increases, or decreases [75]. In addition, a novel α-gliadin was detected. As a compensatory effect of the reduction in gliadins, levels of high-molecular-weight glutenins increased, resulting in a lower gliadin-to-glutenin ratio in the GMP compared to the wild type. Simultaneously, higher lysine contents (an increase of up to 67%) were also observed, which is likely due to a compensatory increase in high-molecular-weight (HMW) glutenins, albumins, and globulins [105]. Some authors observed changes in the protein body morphology of those transgenic lines, in which all gliadins were downregulated [104].

#### 3.1.2. Plants Producing Novel Oils in Seeds

The production of novel substances such as long-chain ω-3 fatty acids, specifically docosahexaenoic acid (DHA), and eicosapentaenoic acid (DPA), which are also referred to as fish oils, in seeds of oilseed crops requires the introduction of a novel metabolic pathway derived from other organisms, usually microalgae. The expression of several novel enzymes in the crop plant is needed to convert the available substrates, i.e., oleic acid, linoleic acid, and α-linoleic acid, into the targeted ω-3 fatty acids, e.g., via the δ-6 desaturase pathway [35,80,106]. The new enzymatic activities in the GMP affect the overall fatty acid composition and results in the production of several novel intermediate fatty acids (e.g., stearidonic acid (SA) and γ-linolenic acid (GLA)) in seeds. After successful modification, the GMPs produce specific types of ω-6 and ω-3 fatty acids, which are neither produced in wild-type plants nor detectable in fish oil [76,77,80,106]. Depending on the approach, these intermediate fatty acids were in the range of 2–3% of total fatty acids in *Camelina* sp., but much higher (e.g., 27% GLA and 5% SDA) in other plant species [76,77,107,108]. The unintended intermediate fatty acids occur in the plant at various levels, depending on the efficiency of the newly introduced enzymatic steps needed to synthesize the targeted fatty acids [76,109].

#### 3.1.3. Plants Producing Pharmaceuticals or Nutraceuticals

Medicinal herbs producing pharmacologically relevant plant metabolites such as natural antioxidants (e.g., phenolics), pigments (e.g., carotenoids), plant defense substances (e.g., flavonoids), alkaloids, and other aromatic compounds are genetically modified to change the endogenous level of these therapeutic phytochemicals, and are referred to as “pharmaceuticals” or “pharm crops” (see reviews in [88,110,111,112,113,114]). An example is the modification of the benzylisoquinoline alkaloid (BIA) pathway in opium poppy, *Papaver somniferum* [83]. By using CRISPR/Cas, several of the plants’ endogenous alkaloids (e.g., morphine and codeine) were significantly reduced. Simultaneously, a novel uncharacterized alkaloid was detected [83].

In addition, so-called biofortification approaches target the expression of phytonutrients in plants, with the aim of achieving an improved nutritional profile; these are called nutraceuticals (see overview in [32]). One example for improving the nutritional value of crops is the expression of the health-promoting carotenoid astaxanthin, which is usually not synthesized in plants and is only available from fish, shrimps, and microorganisms. Zhu et al. [87] achieved the synthesis of astaxanthin in rice plants through the heterologous expression of several synthetic gene expression cassettes. In addition to the targeted astaxanthin, the transgenic lines expressed various carotenoids and carotenoid intermediates in the endosperm of the rice grains, which are usually absent in wild-type plants [87].

#### 3.1.4. Plants Producing New-to-Nature Compounds

By engineering novel metabolic pathways into plants, new-to-nature compounds can be produced that have not been detected in wild-type species so far. For example, Calgaro-Kozina et al. [89] developed a new-to-nature biopesticide by transferring a glucosinolate pathway from *Brassica rapa* into tobacco *N. benthamiana*. The plants produced new types of phytoalexin molecules, which were described as “crucifalexins” by the authors. By expressing two to three heterologous enzymes simultaneously, different abundances of the novel crucifalexin molecules were detected, while the production of the naturally occurring molecule brassinin was stopped (see Figure 1c in [89]). The authors further optimized the novel pathway in the GMP and transferred additional bacterial enzymatic genes to the plant. As result, two novel biomolecules, chlorobrassinin and bromobrassinin, were described. Some of these showed better inhibitory activity against a cruciferous pathogen than their natural analogous molecule brassinin and were comparable to a commercial pesticide in its efficacy.

#### 3.1.5. De Novo Domestication and Re-Domestication of Plants

New genomic techniques in plant breeding are harnessed for the de novo domestication of plants by breeding key domestication traits into wild crop progenitors or wild plants (see overview in [97,115,116,117,118]). The domestication of crops has often involved a loss of genetic diversity and beneficial traits such as biotic or abiotic stress tolerances. Many of these traits are difficult to cross-breed into modern varieties. In contrast, genes that are important for crop domestication are well described and accessible through the application of NGTs [116,119]. Zsögön et al. [90] and Li et al. [91] used genome editing to create de novo domesticated tomato plants derived from the wild relative plant of tomato, *Solanum pimpinellifolium*. This species is resistant to bacterial spot disease and is also salt tolerant; these are considered beneficial traits in tomato. The simultaneous modification of several genetic loci in this wild tomato plant resulted in a range of novel plant phenotypes with a more compact plant architecture and different morphology compared to the wild plants, while still retaining the beneficial traits of the parental wild relative [90,91]. Comparing the novel GM phenotype with the wild type resulted in significant differences, specifically in common plant characteristics, which are usually assessed during the agronomic and phenotypic characterization carried out in field trials for the comparative assessment in ERA. For example, fruit weight in de novo domesticated tomato increased to 200% compared to the wild type. The time to flowering (leaves to first inflorescence; see Figure 1e in [91]) was significantly reduced. Flower number increased, while plant height decreased significantly due to the bushy morphology of the novel phenotype compared to wild tomato [90]. At the same time, the biotic or abiotic stress tolerance of the wild parental plant was retained (for details, see [90,91]). The concentration of the carotenoid lycopene, which is an important determinant of the nutritional value of tomato [120], was 100% higher in the novel GM phenotype than in the parental wild type, and was more than 500% higher than in conventional commercial cherry tomatoes [90], thereby exceeding any natural variation in cultivated tomato varieties known so far. Non-equivalences between the GMP and conventional tomato plants are also likely with respect to the presence of secondary metabolites, which are present in the wild parental plant (e.g., alkaloids or glucosinolates) and which significantly exceeded common levels in food crops. These can affect human health but also the environment, e.g., biotic stressors [115]. In addition, unexpected epistatic effects in the modified phenotype are not unlikely if newly introduced alleles have already played a role in the domestication process [121]. In general, there is still a lack of knowledge about the interconnectedness of the metabolic networks of the many genes that contribute to a desired domestication trait [122].

In addition to the wild relatives of domesticated crops, orphan crops, crop-wild relatives, or weeds, including perennial plants, have been proposed for de novo and re-domestication [93,117,118,123]. Orphan crops (e.g., pseudo cereals, grain amaranth, buckwheat, legumes, or root crops) are considered minor or neglected crops, which are cultivated locally or in limited areas and have often not been fully domesticated. Lemmon et al. [92] modified the genes of production-relevant traits such as plant architecture, flower production, and fruit size in the orphan crop “ground cherry” *Physalis pruinosa*, a plant species that is commercially grown in Central and South America [124]. De novo domestication was also carried out in wild rice *Oryza alta* [94], as well as semi-wild (or semi-domesticated) rice *Zizania latifolia*, to re-introduce a lost ancient cereal crop [95]. In addition, weeds like *Echinocloa crus-galli* or *E. oryzicola*, which are phenotypically similar to cultivated rice but different in seed shattering, have also been used for de novo domestication [95]. These plants have no or little history of cultivation and—if de novo domesticated—do not only differ in morphology and composition from familiar food crops but also with respect to their ecological characteristics and agricultural performance. As these plants tolerate unfavorable environmental conditions or are able to persist in fields for long periods of time, their cultivation methods (e.g., fertilizer need and tillage) will likely differ from those of commonly grown food crops.

#### 3.1.6. Plants with Enhanced Photosynthesis (“Green Carbon Plants”)

With NGTs, the engineering of CO_2_ fixation in the plants and natural pathways involved in the photosynthesis of plants (e.g., photorespiration and light-harvesting) has become possible, with the aim of increasing photosynthetic efficiency, carbon fixation, and crop yield. In addition, synthetic biology enables the design of alternative, new-to-nature pathways to modify photosynthetic metabolism, as evidenced in bacteria so far [125]. These complex GMP applications have also been termed “green carbon plants” (for review, see e.g., [99]). Applications aiming to engineer natural CO_2_ fixation pathways are still in the proof-of-concept stage in model plant species; knowledge on potential unintended changes in these GMP is currently lacking. While the central photosynthetic metabolism is governed by around 100 genes, another 3000 genes are involved and relevant for the phenotypic variation in photosynthetic efficiency observed in plants [126]. As many other metabolic pathways contribute to the photosynthetic properties of plants (e.g., water use efficiency or nutrient use efficiency), tradeoffs between these parameters are likely and may considerably affect the overall plant phenotype, particularly the plant’s development and growth.

### 3.2. Intended and Unintended Changes Are Blurred

In conventional GM crops, the intended trait is easy to identify as it usually refers to a single or a few newly expressed proteins (e.g., the introduced EPSPS or Cry protein). In complex GM applications, however, it is often difficult to demarcate the intended trait from a range of other co-occurring changes in the plant phenotype. Several examples for such collateral effects of genetic modification are evident in complex GMPs (see Table 1 and Section 3.1.), blurring the line between the intended trait and unintended phenotypic changes.

For example, modifications of fatty acid metabolic pathways often result in changes in the relative frequency of various fatty acids, therefore leaving room for interpretation with respect to which changes were intended and which were unintended. Even though the original intention of the modification, e.g., high levels of oleic acid or the expression of LCPUFAs in plants, was achieved, other observed changes were actually not intended, such as the observed accumulation of novel or intermediate fatty acids. Similar observations can be made when the seed protein metabolism is modified, changing the ratio of different gluten components, in addition to unintended changes in other seed metabolite levels (Table 1). Depending on the specific approach used to achieve a complex GM trait, the unintended phenotypic changes can differ. In order to synthesize LCPUFAs such as EPA and DHA in oilseed crops, the δ-6-pathway (the conventional aerobic pathway) is frequently modified. Several alternative pathways exist (e.g., the PKS-like pathway) to synthesize DHA and EPA. All pathways finally result in the accumulation of LCPUFAs in plant seeds but with varying levels of effectiveness and occurrences of biosynthetic intermediates [77,127].

### 3.3. Lack of Familiarity and Presumed Safety of the Parental Plant

With new genomic techniques, the range of plant species addressed by genetic modification is broadened beyond the crop species used for classic genetic modification [2,128]. For complex GMP applications, in which the parental plant is a non-familiar plant or a non-crop species, the validity of the familiarity and HoSU concepts (see Section 2.2.) and the presumed safety of the parental plant must be questioned. Today, plant species are used for genetic modification and genome editing, which are cultivated only sporadically, such as *Thlaspi arvense* [70,72,73,129], *Crambe abyssinica* [82], or *Lepidium campestre* [71,130]. Orphan crops, which are also increasingly targeted for re-domestication, are often locally important staple crops grown in developing countries [95]. These plants have no cultivation history in Europe and knowledge regarding their agronomic performance in the EU is limited (e.g., for *Solanum pimpinellifolium*, see [131]). Some plants are considered as weeds, such as *Thlaspi arvense* or *Echinochloa crus-galli*, for which no familiarity or HoSU exists at all. Genetically modified weeds, if used in a novel receiving environment, are likely to entail environmental risks, e.g., with respect to persistence and invasiveness, that are analogous to non-native species. If considered as invaders elsewhere, they might be classified as invasive species in the EU. Weeds, as well as wild plants in general, but also orphan crops, have a higher phenotypic plasticity and are generally more adapted to extreme soil and climatic conditions [132]. This plasticity in response to nutrient and water availability under agricultural conditions can relate to many different agronomically relevant traits such as plant height, leaf area, photosynthetic rate, and growth performance traits, but may also affect reproductive plasticity [133,134]. The lack of experience with these plants does not only refer to their performance and management within agricultural fields but also outside the agricultural context (e.g., in semi-natural habitats). Hence, not only their novelty in cultivation and use, but also their novelty outside agro-ecosystems—with potential adverse consequences for biodiversity and the environment—must be taken into account in ERA.

The OECD publishes guidance documents on the biology or composition of crop plants, providing background data for the safe use of GM crop plants, such as maize, wheat, canola, soybean, rice, and potato [135]. The lack of knowledge about the safe use of non-crop plant species shows that the concepts of HoSU and familiarity cannot be applied to species other than familiar crop plants in ERA. Consequently, the hitherto applied concepts of familiarity and HoSU are not practical for the ERA of complex modifications in non-crop plants.

### 3.4. Unclear Biological Relevance of Observed Differences and Lack of Safety Limits

In current ERA practice, observed (phenotypic, agronomic, and compositional) differences between a GMP and its non-GM parental plant are put into context with the observed variability of non-GM plants (i.e., reference varieties). In the case of observed differences and non-equivalences, the relevance for food and feed safety was so far based on the HoSU concept, as well as on expert judgment, to assess the ability to cause harm [136]. For the assessment of environmental effects (e.g., effects of the GMP on non-target organisms), other authors considered this approach inappropriate as it lacks safety limits [61].

In contrast to classical GM crops, a range of non-equivalences between the GMP and the reference varieties are expected to occur with complex GMP applications (see Section 3.1.). With regard to the biological relevance of observed differences for the environment, the particular protection goals must be taken into account [45]. The comparative analysis of nutrients and anti-nutrients clearly relates to the protection of the health of humans and animals. For example, in the case of the high-oleic-acid soybean (event 305423), EFSA considered the increased consumption of odd-chain fatty acids, the low levels of polyunsaturated fatty acids (PUFAs), and the increase in mono-unsaturated fatty acids (MUFAs) “small and without impact on health and nutrition” [60]. For plants producing fish-oil-like LCPUFAs, such as DHA and EPA, EFSA has defined safety limits for their use as feed ingredients to protect human and animal health [137]. The tolerable levels for food safety are based on the toxicological and nutritional impact of the individual fatty acids [137]. However, such limits are lacking for environmental safety, due to the limited knowledge of the role of specific metabolites in the environment.

A range of plant metabolites with relevance to environmental safety has been described for common crop plants, particularly entomotoxic proteins in seeds (e.g., protease and amylase inhibitors or lectins; see overview in [138]). In vegetative plant tissues, compounds like a naturally occurring benzoxazinoid (e.g., DIMBOA) are considered to be associated with toxicity to insects [139]. However, these metabolites are—by default—not included in the comparative compositional analysis, and safety limits for these substances are lacking. This lack equally applies to novel plant metabolites and compounds targeted in complex GMP applications.

For changes in plant endogenous metabolites in medicinal herbs that act as natural antioxidants (e.g., phenolic compounds), natural pigments (e.g., carotenoids), plant defense substances (e.g., flavonoids), or metabolites such as alkaloids and aromatic compounds [110,111,112], knowledge about their functional relevance for ecosystems is available [140] but safety limits are also lacking. Specifically, the modification of plant-endogenous alkaloids through the use of CRISPR/Cas (see Table 1) can have consequences for terrestrial food webs due to their importance for plant–insect interactions [141]. For completely novel substances such as new bio-pesticides or new-to-nature substances, knowledge on safety limits for both food and environmental safety does not exist. In addition, such novel substances severely challenge established concepts of protein safety assessment, as recently recognized by EFSA in a draft scientific opinion [142].

## 4. Consequences for the ERA of Complex GMP Applications

### 4.1. Importance of a Protection-Goal-Focussed ERA

The requirements for a comparative safety assessment approach are no longer met for new plant phenotypes such as those developed by metabolic engineering or de novo domestication. The identified shortcomings and limitations call for a hypothesis-driven approach that complements a comparative assessment of the GMP with non-modified plants. A hypothesis-driven assessment approach focuses on the potential adverse impacts of the GMPs on valued entities and specific protection goals for each area of risk rather than drawing risk conclusions based on the differences and non-equivalences between the GMP and other (non-GM) plant varieties. Formulating risk hypotheses in ERA integrates potential risks of the parental plant, the intended GM traits, any unintended (e.g., metabolic or phenotypic) changes in the plant, and the (potentially novel) receiving environment (Figure 1b). This approach is based on the recognition that the hazard may be composed of the whole novel plant phenotype instead of searching for hazards only based on differences in single, standardized plant characteristics. This is also in line with the original definition of a hazard in ERA, which refers to the potential of an organism to cause harm to human health or the environment [143].

In order to carry out a hypothesis-driven ERA, specific protection goals that may be affected by a specific GMP application in different receiving environments and related assessment endpoints should be defined at the start of the problem formulation. For human health and food safety assessments, toxicity or allergenicity are suitable and accepted assessment endpoints for the protection goal human health [144]. For environmental risks, protection goals are more general and should consider effects on biodiversity and agro-ecological functions including sustainable land use [24]. The protection goals and assessment endpoints need to be specified for the major risk areas, as outlined by Directive 2001/18/EC and EFSA [24], not only including risks to non-target organisms but also risks, e.g., due to the persistence and invasiveness of the GMP. The EFSA has developed an approach to translate the general goals into operational and specific ones (specific protection goals—SPGs) based on the ecosystem service concept [28]. These are considered applicable for different environmental stressors such as pesticides, feed additives, or GMPs [28,145,146]. So far, such SPGs have been discussed only for non-target Lepidoptera in the context of *Bt* maize pollen exposure [28].

**Figure 2:**
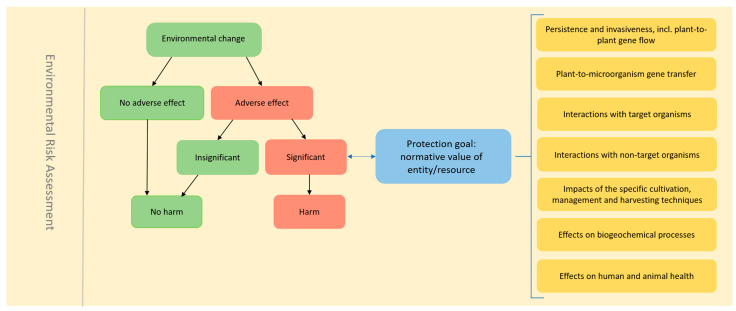
Pathways to environmental harm in environmental risk assessment of complex GMP. Environmental harm occurs when a protection goal is adversely and significantly affected by various types of effects outlined in [24]. Modified after [149]. See text for further explanation.

Earlier, Kowarik et al. [147,148] and Bartz et al. [149] proposed a concept for the operationalization of environmental harm in the context of GMO ERA. They suggest linking policy goals and normative harm levels with descriptive effect levels for the definition of environmental harm in ERA. This approach involves defining significant adverse effects on a valued entity or conservation resource that can be distinguished from a non-significant or non-adverse effect. The normative value, such as the conservation status of the entity or conservation resource, is a key element in determining the difference between insignificant and significant adverse effects (Figure 2). We propose that the definition of the significance of an adverse effect has to be contextualized within the relevant protection goal for each risk area, as outlined by EFSA in its guidance document [24].

### 4.2. Potential Environmental Risks of Complex GMP Applications

#### 4.2.1. Risks for Trophic Food Webs and Biodiversity

Complex GMP applications with modifications of metabolites in seeds (see Case Study 1; Table 1) may affect trophic food webs and biodiversity through the observed shifts in the relative frequency of a plant’s endogenous substances and metabolites (e.g., diverse fatty acids or gluten components). Such shifts have unknown implications for plant-feeding organisms (e.g., herbivores, granivores, or pollen-feeding insects). Specifically, ω-3-PUFAs are essential in the diet of many animals and can affect a range of physiological performance variables in seed-feeding animals, e.g., in birds [150,151,152]. Koller et al. [101] discussed the potential risks of imbalances in PUFA diets for pollinators feeding on the pollen of oil-modified Brassicaceae.

The expression of novel substances and molecules in plants (e.g., fish oil fatty acids, specific types of carotenoids, and novel biopesticides) that are so far not produced in terrestrial ecosystems (Table 1; Case Studies 2 and 4) can also affect plant–herbivore interactions, possibly leading to changes in food webs and biodiversity. For example, the expression of fish oils (EPA and DHA) in plants is a new source of LCPUFAs in agro-ecological environments with unknown effects on herbivores, granivores, and other non-target organisms. Such adverse effects have been evidenced in crop pests that showed developmental deficiencies upon feeding [109,153,154,155]. Other metabolites that are newly expressed in biofortified GMPs (Table 1; Case Study 3), such as astaxanthin [87], are usually synthesized in microalgae or bacteria, and may also have unexpected consequences in agro-environments. While astaxanthin has a high nutritional value for a variety of higher organisms including humans [156], there is currently little evidence regarding its effects on terrestrial insects. However, some beneficial effects of this carotenoid have been observed in terrestrial spider mites [157].

Potential risks for biodiversity may also derive from the heterologous overexpression of therapeutic phytochemicals in “pharm crops” such as different structural classes of alkaloids, which are known to exhibit adverse effects on herbivores and can also affect plant–insect interactions [141]. Engineering new-to-nature plant metabolites in plants, such as the newly described class of antifungals—crucifalexins—in Brassicaceae [89] or new and uncharacterized types of alkaloids in opium poppy [83] (Table 1; Case Study 4), may negatively impact a range of non-target organisms such as pollinators, which is comparable to toxic alien biomolecules produced by invasive alien species [158,159]. Further research is therefore required to elucidate both their functional role in the ecosystem and their potential for adverse impacts before safety limits can be defined.

#### 4.2.2. Risks Due to Changes in Stress Tolerance and Plant Survival

Plants with alterations of the fatty acid profile in seeds (Table 1; Case Studies 1 and 2) may be severely impaired under stress conditions [153]. It is known that fatty acids play an essential role in cell membranes and as hormone precursors in plants [160] and are therefore relevant to a plant’s biotic stress response. For example, the fatty acid linolenic acid, which is a precursor for the synthesis of LCPUFAs in complex GMPs, also acts as a precursor of jasmonic acid, which is a plant hormone that is relevant for stress response and development [70]. In addition, in the cell membranes of plants, the degree of saturation of fatty acids influences membrane fluidity and, thereby, stress tolerance [161]. The expression of specific desaturase enzymes in GMPs changing fatty acid saturation to achieve the synthesis of LCPUFAs [76,77] may therefore affect stress tolerance in these plants.

Fatty acids including LCPUFAs also play an important role in seed germination and seedling development [162]. In GM approaches engineering waxy esters in seeds, negative effects on seed weight or seed germination and early plant growth have been observed [81,82]. Other approaches targeting fatty acid composition in seeds also showed adverse effects on seed germination, seed size and morphology, seed oil content, and seedling development, at least under experimental conditions [66,69,70,73,110,163]. Some of these effects have been explained by the slower rates of mobilization of PUFAs present in seeds, but also by lower specificities of the seed lipases for the specific fatty acids available in the seed (see discussion in [162]). The intended mutagenesis of seed coat color in plants unintentionally modified PUFA levels [74], thereby possibly also affecting the germination characteristics of the seed. However, it is currently unknown whether the observed effects have implications for seed survival (e.g., dormancy) or plant establishment and thereby the persistence and invasiveness of these GMPs under natural conditions.

#### 4.2.3. Risks Due to Persistence and Invasiveness

So far, crop plant species have been used for genetic modification; these plants are familiar and are, thus, largely predictable with regard to their environmental behavior in and outside agricultural fields, with regard to, e.g., volunteer formation, feralization, or hybridization with wild relatives (e.g., maize, soybean, and oilseed rape). For plants that have never or rarely been cultivated under European agricultural conditions (e.g., wild plants or completely novel plant phenotypes), similar experiences are not yet available, and their environmental performance, i.e., with respect to survivability or persistence in and outside agricultural habitats, is less predictable (Table 1; Case Study 5). For example, *S. pimpinellifolium*, the wild relative of tomato, is considered an invasive or alien species in many tropical and temperate countries with few records of its occurrences in Central Europe, which may stem from difficulties in morphologically distinguishing cultivated tomato *S. lycopersicum* from wild tomato [164,165,166]. Difficulties in morphological distinction between species may apply for most of the de novo domesticated wild or weedy species [123].

In addition, GMP applications with improved carbon fixation pathways (Table 1; Case Study 6) may have a competitive advantage over non-GM plants, which may increase their competitive ability and affect their persistence or invasiveness. Photosynthetic capacity is a determinant for competitive success, e.g., in weedy species, and can influence the invasion success of non-native plants under certain conditions [167,168,169].

### 4.3. Development of Test Systems for Assessing the Environmental Performance of New Phenotypes

For non-familiar GMP phenotypes, ERA can no longer rely on the presumption of familiarity and the safety of the parental plant, but should consider the whole plant phenotype and its environmental performance in the receiving environments. The assessments of single agronomic or compositional characteristics of the GMP in field trials following a standard experimental setup [24,43], as is currently being conducted in the comparative safety assessment, is of limited value for drawing conclusions regarding the environmental risks of such GMPs. We therefore call for new assessment approaches based on experimental settings that reflect potential changes in the environmental performance (e.g., germination and/or survival) of the novel phenotype in their respective (and potentially novel) environments. In addition to the extension of the standard seed germination tests that are currently used in ERA [62], we advocate for additional experimental approaches to assess the survival and competitiveness of plants, e.g., through the use of competition experiments or fitness assessments.

Plant competition and fitness assessments need to be carried out in experimental setups separately from the standard agronomic field trials used for comparative assessments in ERA. Such manipulative experiments, carried out under contained (greenhouse) or field conditions, are common in plant ecology research and have already been conducted with a range of GMPs, such as soybean, oilseed rape, sunflower, rice, or maize (for an overview of available studies, see [170]). Competition studies investigating the competition ability of (usually) two plant taxa, e.g., a GMP and a wild competitor, can be conducted in the greenhouse or under field conditions (see, e.g., [171,172,173,174,175,176]). Plant fitness assessments have been conducted in the context of GMP risk assessment, e.g., for assessing the relative fitness of crop–wild hybrids [173]. Experiments in greenhouses or growth rooms with plants in pots (e.g., [177,178,179,180]) or mesocosms similar to small field plots have been carried out [181,182]. In addition, there are approaches combining contained and field assessments to make conclusions about the fitness or competitiveness of the GMP (e.g., [177,183]). Such experiments are in line with additional measurements recommended in the context of agronomic assessments for persisting species or species with feral populations [43]. However, so far, the selection of separate and specific experiments to test risk hypotheses is currently left to the applicant. We therefore suggest the development of further guidance with respect to appropriate methodological approaches and the interpretation of results by, e.g., EFSA.

Considering the whole new GM plant phenotype in an ecologically relevant experimental setting will take account of all (intended and unintended) changes in the GMP due to the genetic modification and potential adverse environmental consequences thereof. Such an assessment approach thereby supports the generation of risk-relevant data in ERA.

### 4.4. Adaptation of the Comparative Assessment of Standard Agronomic and Phenotypic Traits

An important component of the comparative safety assessment approach is the assessment of agronomic and phenotypic differences between the GMP and its comparators assessed in agronomic field trials by use of standard plant parameters. The aim of this standard assessment is to test whether the genetic modification has (unintentionally) changed the phenotype of the GMP [184] and to characterize changes in the basic biological and agronomic traits of the GMP. These standard assessments should not be used to derive conclusions on the environmental safety of the GMP [62]; however, an assessment of those parameters (i.e., phenotypic plant characteristics) that are relevant for a specific risk hypothesis is useful and practicable. We therefore suggest reducing the extent of the comparative assessment for complex GMPs. This avoids the currently applied unspecific phenotypic profiling of the GMP in comparative assessments with unknown biological relevance of their outcomes [185]. For example, focusing the compositional assessment to specific toxicants or anti-nutrients in the seed or vegetative tissues of the respective plant taxon can provide information on potential risks for herbivorous non-target organisms, e.g., in the case of plants producing pharmaceuticals or nutraceuticals. Similarly, focusing the agronomic assessment of a novel plant phenotype on potentially changed germination and seedling establishment characteristics can provide information on the potential of the plant to survive under a range of different conditions, including sub-optimal conditions (e.g., drought). This could be achieved, for example, by assessing the performance of the plant under standard agricultural conditions but also under conditions without management measures (e.g., no fertilizer or irrigation) or by experimentally applying specific stress conditions (e.g., drought). Taking sub-optimal growing conditions into consideration during the agronomic and phenotypic characterization of the GMP can indicate the plants’ potential to germinate and survive under less-than-optimal conditions, e.g., when spreading to and establishing in semi-natural habitats (see [62] for further discussion). This is particularly relevant for complex GMPs for which the parental plant has no familiarity and history with regard to cultivation or growth in and outside EU ecosystems (e.g., green carbon plants or de novo domesticated crops).

## 5. Conclusions and Future Outlook

The provisions for ERA of GMPs, according to Annex III of Directive 2001/18/EC and the ERA guidance from EFSA [24], are applicable to higher plants of the taxonomic group Spermatophytae, which are mostly land plants comprising approximately 270,000 plant species. Fewer than 200 plant species are cultivated today for food purposes, with only nine species providing the majority of the human food supply [186]. So far, about 25 genetically modified plant species (mainly for food and feed purposes) have been risk assessed [187]. With NGTs, the scope for modifying plant species is expanding, increasing not only the depth of intervention on the genetic level but also achieving fundamental systemic and metabolic processes in plants. Genetic modifications have now also become feasible for a broad spectrum of non-crop plants, including crop–wild relatives or weeds [188]. This challenges the current ERA, which has been developed for the assessment of genetically uniform and familiar crop plants in managed agro-ecosystems.

The regulatory initiative started by the European Commission to deregulate plants obtained by certain types of NGTs and to exclude them from current risk assessment requirements [189] addresses not only crop plants but refers to a large group of plants with a lack of experience in cultivation and use, including wild species. This deregulation could be a potential threat to natural ecosystems, as NGT plants, for which risks can be identified, would neither be risk assessed nor monitored. In such cases, changes in the persistence and invasiveness of NGT plants may cause problems similar to those posed by invasive species, for which damage to biodiversity and ecosystem services, as well as economic costs related to their management, are well documented [190,191]. National risk assessment authorities in the EU have recognized the lack of knowledge on environmental risks for plants other than crops if modified by NGTs [192], underpinning the need to consider environmental risks in the European Commission’s initiative on NGT plants.

Therefore, we recommend deriving conclusions on the environmental safety of novel and non-familiar complex phenotypes based on an assessment approach that focuses on the potential risks of the GMP linked to protection goals. Such an approach integrates the effects of the parental plant and novel traits, as well as any systemic effects caused by the genetic modification. To achieve this, we suggest defining a potential hazard based on evidence from the entire GM plant (and potentially secondary stressors). For complex genetic modifications, hazards can no longer be narrowed down to single phenotypic differences between the GMP and the non-GM plant. Experimental assessments should be based on specific risk hypotheses for the different risk areas outlined in ERA guidance [24]. Therefore, we suggest updating current ERA methodologies to include ecologically more realistic whole-plant assessments and putting less focus on standard field trials with prescribed endpoints and plant characteristics. Risk hypotheses will diverge considerably between complex GMP applications. The appropriate assessment approaches are likely to be more complex compared to current assessments due to the variety and diversity of GM plant species that are about to approach the risk assessors’ tables in the near future.

We expect that compared to classical GM crops, the ERA for complex GMP applications will require a stronger focus on risks due to persistence and invasiveness (including plant-to-plant gene flow). Hitherto, ERA is mostly informed by reference to historical and current cultivation experience and knowledge on the environmental behavior of familiar crop plants in EU environments like maize or soybean. Due to a lack of experience with GMP cultivation in the EU in general, no post-market monitoring data are available, which are necessary to validate risk assessments and their outcomes. The only exception is *Bt* maize, for which risk assessment conclusions for persistence and invasiveness were re-evaluated, and management measures were updated, due to the emergence of a noxious weedy relative—teosinte [193,194]. This shows the importance of this risk area being addressed in ERA, which will gain importance with new plant taxa and novel GM traits. Importantly, assessment approaches covering risks due to persistence and invasiveness need to extend beyond the managed cultivation area and optimized growing conditions. To achieve this, further and specific guidance is needed for this risk area, analogous to the existing guidance for the assessment of potential impacts of GMPs on non-target organisms [25].

Efficient and practicable ERA should avoid collecting and evaluating data for which the biological relevance is uncertain. This implies that assessment concepts and decision criteria need to be established at the same time that methodologies and experimental designs are drafted. In the past, the assessment and interpretation of comparative data sets remained unclear. In order to increase confidence in risk conclusions in ERA, policy objectives have to be translated into specific protection goals, relevant risk hypotheses, and harm thresholds. For GMPs with complex modifications, this can only be achieved by an additional hypothesis-driven and protection goal-oriented assessment approach that complements the comparative safety assessment approach used to date.
